# A population of descending neurons that regulate the flight motor of Drosophila

**DOI:** 10.1016/j.cub.2022.01.008

**Published:** 2022-01-31

**Authors:** Shigehiro Namiki, Ivo G. Ros, Carmen Morrow, William J. Rowell, Gwyneth M. Card, Wyatt Korff, Michael H. Dickinson

**Affiliations:** 1 Janelia Research Campus, Howard Hughes Medical Institute, 19700 Helix Dr., Ashburn, VA 20147, USA; 2 Division of Biology and Bioengineering, California Institute of Technology, 1200 E. California Blvd., Pasadena CA, 91125, USA; 3 The University of Tokyo, Research Center for Advanced Science and Technology, 4-6-1 Komaba, Meguro, Tokyo 153-8904, Japan; 4 Contributed equally to manuscript.; 5 Lead Contact

## Abstract

Like many insect species, *Drosophila melanogaster* are capable of maintaining a stable flight trajectory for periods lasting up to several hours^1,2^. Because aerodynamic torque is roughly proportional to the fifth power of wing length^3^, even small asymmetries in wing size require the maintenance of subtle bilateral differences in flapping motion to maintain a stable path. Flies can even fly straight after losing half of a wing, a feat they accomplish via very large, sustained kinematic changes to both the damaged and intact wings^4^. Thus, the neural network responsible for stable flight must be capable of sustaining fine-scaled control over wing motion across a large dynamic range. In this paper, we describe an unusual type of descending neuron (DNg02) that projects directly from visual output regions of the brain to the dorsal flight neuropil of the ventral nerve cord. Unlike many descending neurons, which exist as single bilateral pairs with unique morphology, there is a population of at least 15 DNg02 cell pairs with nearly identical shape. By optogenetically activating different numbers of DNg02 cells, we demonstrate that these neurons regulate wingbeat amplitude over a wide dynamic range via a population code. Using 2-photon functional imaging, we show that DNg02 cells are responsive to visual motion during flight in a manner that would make them well suited to continuously regulate bilateral changes in wing kinematics. Collectively, we have identified a critical set of DNs that provide the sensitivity and dynamic range required for flight control.

## eTOC BlurB

Using an activation screen in flying flies, Namiki et al. identify a population of descending neurons that regulates wing amplitude over a large dynamic range. Via functional imaging and activation of different numbers of cells, they show that this population is a core component of the flight circuit, allowing the fly to steer and fly straight.

## Results

**Figure 1.**
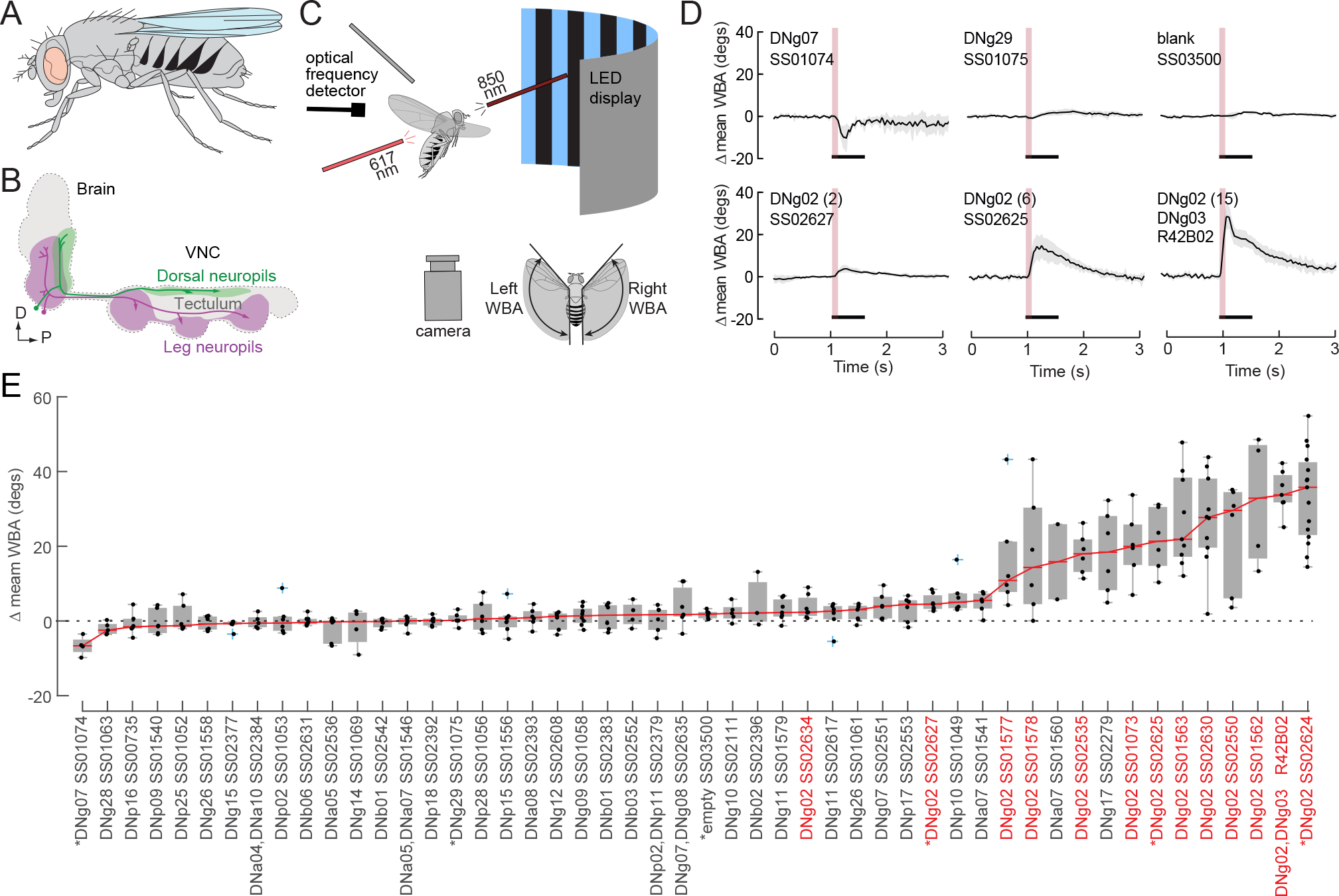
Experimental methods for optogenetic activation and screen results. (A) Cartoon showing location of central nervous system within a fly. (B) Descending neurons (DNs) are stratified into two main groups: a ventral group (magenta) that innervates the three leg neuromeres and a dorsal group (green) that innervates the dorsal neuropils associated with the neck, wings, and halteres. (C) Cartoon showing experimental set-up (not drawn to scale). A fiber optic cable delivering 617 nm light is positioned behind a tethered fly aimed at the thorax. Two fiber optic cables (only one is shown) deliver continuous near-IR light (850 nm) to illuminate the left and right stroke planes of the fly. The fly is centered within a curved visual display of blue (470 nm) LEDs upon which a static stripe drum (spatial frequency = 36°) was presented to the fly. An image of the fly is captured with an upward facing camera and analyzed using a real-time machine vision system that measures the angular extent of the left and right wingbeat amplitudes. In all experiments, we assumed that the rearward reversal angle was parallel to the body axis and remained constant. An optical detector that recorded fluctuations in IR light was used to record wingbeat frequency. (D) Results of 100 ms pulses of CsChrimson activation on the change in the mean of the left and right wingbeat amplitude of the wings in 6 of the 50 lines tested; red line and gray area indicate the mean response and the standard deviation envelope, respectively. One line (SS01074), which targets the DNg07 neuron, was distinct in that it elicited a consistent decrease in wingbeat amplitude. Most targeted cells, such as the DNg29 neuron shown in Figure 1 (labeled by SS01075) did not elicit a detectable change in wingbeat amplitude. Control flies in which the UAS-CsChrimson line was crossed with an empty vector split-GAL4 line (blank, SS03500) exhibited no response to the 617 nm light. Bottom row: Example traces of different driver lines that target the DNg02 cells. For each trial, we determined the average wingbeat amplitude (WBA) of the left and right wings over the 0.5 second period prior to stimulus onset and subtracted this value from the entire trace to create a zero baseline. The response to optogenetic activation was calculated as the average value of WBA over the 0.5 second period starting with stimulus onset. (E) Overview of the entire screen of 50 lines, ranked from left to right according to the magnitude of the optogenetic effect on wingbeat amplitude. Each trial was scored by averaging the change in the mean of wingbeat amplitude over the 500 ms period beginning with the onset of the light pulse (indicated by black bars in B). For each line, the median response is indicated by a red line. The median value from each individual fly is indicated by a black dot, the box and whisker plots indicate the interquartile range and extreme values for the flies tested; outliers are indicated by blue crosses. Lines that target DNg02 neurons are indicated by red font. Asterisks indicate lines for which the responses are plotted in panel D. In addition to DNg02 and DNg03, the GAL4 driver line R42B02 also targeted several other DNs including DNg07, DNg08, and DNg09. See also Table S1.

Within a fly’s nervous system, sensory information from the brain is conveyed to motor regions of the ventral nerve cord (VNC) by several hundred pairs of descending neurons (DNs) that are roughly stratified into a dorsal pathway that projects to flight motor neuropils and a ventral pathway that project to leg neuromeres^5,6^ (Figure 1A, B). Whereas some DN types exist as single pairs of bilateral cells with unique morphology, other DN types constitute larger populations of nearly homomorphic neurons. To identify classes of DNs that might be involved in flight control, we conducted an activation screen in which we expressed CsChrimson^7^ in one GAL4 line and 48 split-GAL4 lines^8^ that collectively target 29 different DNs innervating the ventral leg neuromeres, the dorsal wing and haltere neuropils, and the tectulum within the VNC^6^ (Figure 1, Table S1), along with one control split-GAL4 line that does not drive expression in any neuron (the “empty” line SS03500). These driver lines were chosen because their relatively sparse expression patterns might lead to interpretable results in the activation screen. In each trial, we aligned tethered, flying flies within a machine vision system to measure wingbeat amplitude^9^. While the flies flew under closed-loop visual conditions in which they controlled the angular velocity of striped drum, we presented brief, 100 ms pulses of 617 nm light to activate the targeted DNs (Fig. 1C, D). In preliminary experiments, we tested seven pulse durations (10 ms, 100 ms, 400 ms, 1 s, 2 s, 4 s, and 8 s), but we chose 100 ms for our screen because this was the shortest duration that consistently elicited a maximum response and we wanted to minimize the possibility of causing synaptic fatigue via repetitive over-stimulation of the neurons. These 50 lines varied not only with respect to the cells they labeled, but also the sparsity of expression. Nevertheless, a clear pattern emerged when comparing the results across lines (Fig. 1E). Of the 13 lines in which CsChrimson activation resulted in the largest change in wingbeat amplitude, 11 targeted members of the same class of neurons, DNg02.

**Figure 2.**
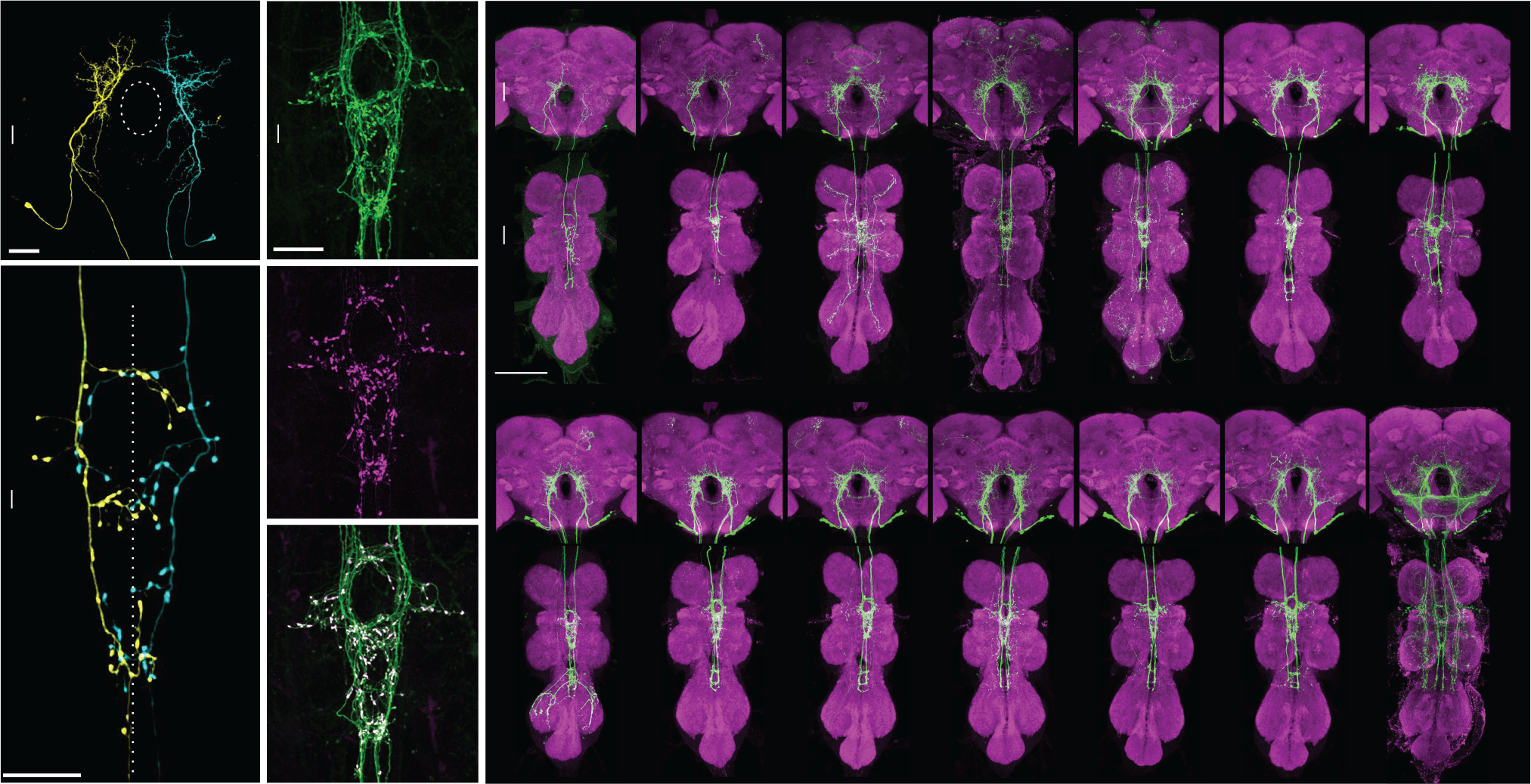
Morphology of DNg02 cells and split-GAL4 driver lines targeting different numbers of neurons. (A) MCFO of SS02627 driver line showing one left and one right DNg02 neuron in the brain; approximate position of esophageal foramen (EF) is indicated by dotted ellipse. (B) Projections of the same neurons in the wing neuropil. The neurons possess punctate terminals on both sides of the VNC midline. (C) Enlarged view of wing neuropil region of SS01578 showing GFP expression in green. (D) Same region as in B, showing synaptotagmin staining in magenta. (E) Merger of images from C and D; DNg02 projections in the wing neuropil contain many output terminals. (F) Expression pattern in 14 different driver lines that target DNg02 neurons. Membrane-targeted GFP expression is shown in green, nc82 staining is shown in magenta. The number of neurons targeted in each line is shown in parenthesis below the line name. See also Figure S1.

The DNg02s were previously identified anatomically^6^ and consist of a cluster of at least 15 cell pairs with morphology that is not easily distinguishable at the level of light microscopy, the largest of the population-type class of DNs identified so far. Their small, spindly cell bodies reside in a cluster at the ventral edge of the gnathal ganglion (GNG; Figure 2A). The primary neurites of the DNg02s run ventrally along the edge of the GNG before taking a hairpin turn and ascending dorsally, where each cell arborizes in a hemi-circle around the esophageal foramen. The cells’ terminals reside within a set of five contiguous neuropils consisting of the inferior bridge (IB), inferior clamp (ICL), superior posterior slopes (SPS), inferior posterior slope (IPS), as well as the gnathal ganglion (GNG)^10^. Synaptotagmin labeling and fine morphology suggest that processes within the IB and GNG are outputs, whereas those within the ICL, SPS, and IPS are inputs^6^.

After descending ipsilaterally down the neck connectives, the DNg02 cells exhibit a distinct pattern of arborization in the dorsal VNC (Figure 2B). Collectively, the population of cells forms a compact ‘figure-of-eight’ shape within the dorsal flight neuropil – a pattern also repeated in the haltere neuropil (Figure 2F). Visualization of the morphology of individual neurons using the multi-color flip out (MCFO) expression system^11^ indicates that the arborizations of DNg02s remain restricted to the ipsilateral side within the brain, whereas the terminals in the VNC cross the midsagittal plane (Figure 2A,B). Large synaptotagmin-positive boutons are distributed diffusely throughout the projections in the wing and haltere neuropils (Figure 2C–E), consistent with output synapses in these regions.

**Figure 3.**
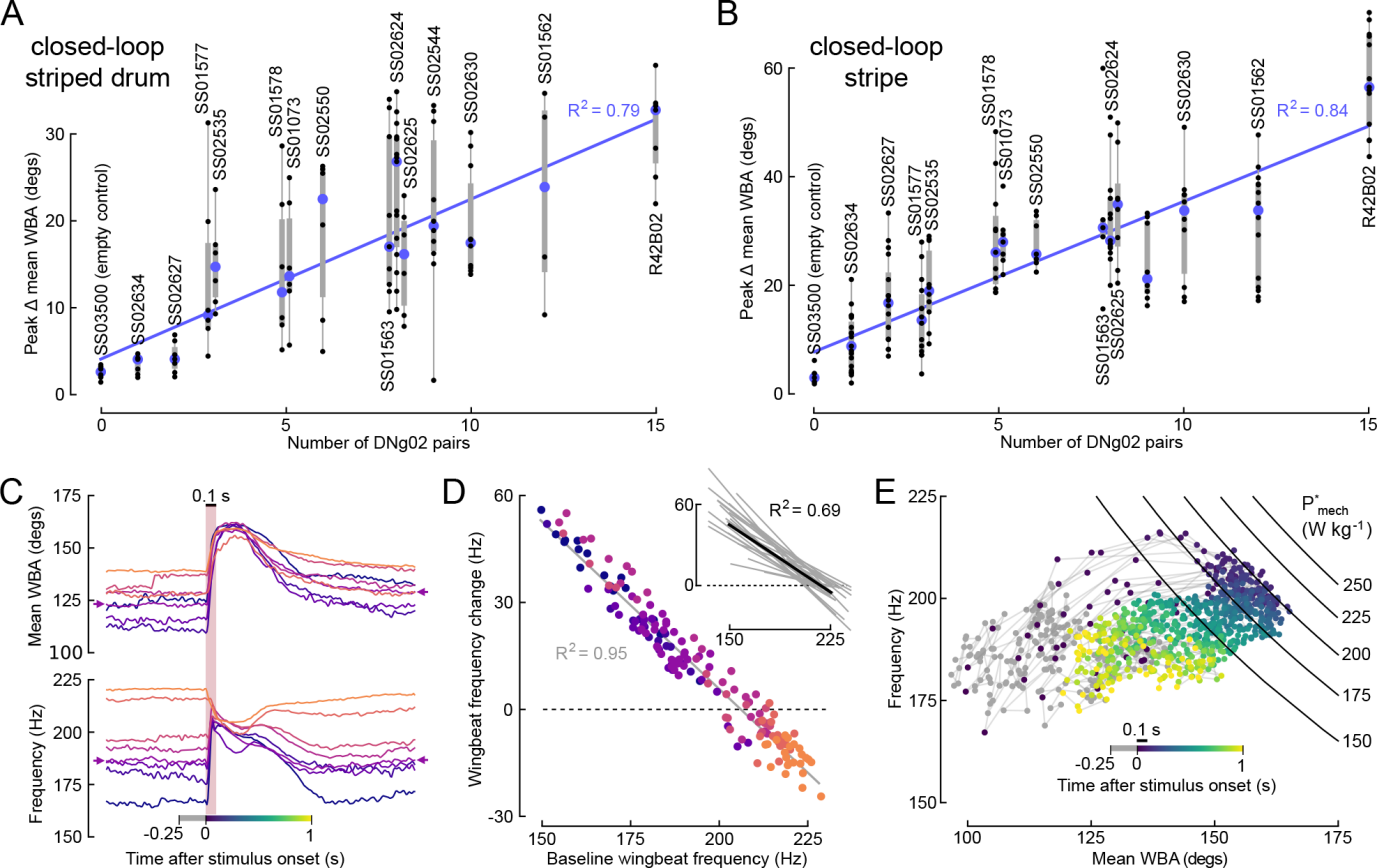
Wingbeat amplitude appears to be regulated via a population code of DNg02 neurons. (A) Peak change in the mean of the left and right WBA elicited by CsChrimson activation in 15 driver lines that target different numbers of DNg02 neurons, replotted from Figure 1D, but now ordered according to the number of DNg02 cell pairs targeted in each line. Blue circles indicate median value for each line, vertical bars indicate interquartile range. The identity of each line is labeled by vertical text above or below the data. Three sets of lines are plotted close together because they target the same number of neurons (SS01577, SS02535: 3 cells; SS01578, SS01073: 5 cells; SS01563, SS02624, SS02625: 8 cells). The number of cell pairs activated correlates positively with the magnitude of the changes in mean WBA (r^2^ = 0.79, based on median values). (B) The results of a second optogenetic screen of the DNg02 lines, conducted under visual closed-loop conditions in which the flies controlled the angular velocity of a dark stripe via changes in the difference in their wing stroke amplitude (r^2^ = 0.84, based on median values). (C) Time series traces for changes in mean ((left+right)/2) wingbeat amplitude (top) and frequency (bottom) elicited by optogenetic activation of DNg02 cells in the SS02625 driver line, from the dataset shown in panel B. Each trace represents the mean response for one fly. Each fly (n=8) is indicated by color, ranked by the level of background wingbeat frequency. Note that whereas the background, pre-stimulus wingbeat angle varied among flies, the peak level elicited by CsChrimson activation was quite similar. Wingbeat frequency also showed a large variation across flies before and after the stimulus, and a decreased variation during activation. Note that wingbeat frequency tends to fall during optogenetic activation following an initial rise. (D) Change in wingbeat frequency during optogenetic activation plotted against the pre-stimulus baseline; color scheme indicates identity of flies plotted in C. Note the inverse relationship; when the background level of wingbeat frequency is above ~200 Hz, optogenetic activation elicits a drop in frequency below baseline. This inverse relationship was extremely consistent across all the driver lines, as indicated by the inset, which plots the comparable regression slopes for all the DNg02 drivers tested in both in the initial (panel A) and second (panel B) screens. (E) Wingbeat frequency plotted against mean ((left+right)/2) wingbeat amplitude for every activation trial of an example fly from panel C indicated by small arrows. For each trial, the time before stimulus onset is indicated by gray, the time after stimulus onset is encoded by color; the duration of the stimulus is indicated by the black bar above the color scale. The same color scale is provided at the bottom of panel C to so that the time course in panels C and E may be compared. At stimulus onset, both wingbeat amplitude and frequency rise; however, after wingbeat amplitude reaches a value of ~160°, further increases are accompanied by a decrease in wingbeat frequency. The black curves show isolines for muscle mass specific mechanical power (P*_mech_) in the frequency-amplitude plane; see text for details.

The large number of DNg02 cells suggests that they might underlie some unique and critical function within the flight motor system. One hypothesis is that the DNg02s act on wing motion via a type of population code^12,13^, such that the precise kinematic output of the wings depends in part on the number of DNg02 cells that are active as well as the level of activity within individual cells. To evaluate this hypothesis, we determined the number of DNg02 neurons that were labelled in each line by counting the number of cell bodies labeled within the gnathal ganglion (Figure 2, Figure S1). When the wingbeat amplitude responses from the optogenetic activation experiments in Figure 1D were replotted according to the number of cell pairs targeted in DNg02 each line, we found a strongly linear relationship (Figure 3A, slope = 1.84 degs per cell pair activated, r^2^ = 0.79). To confirm this result, we repeated our CsChrimson activation paradigm using the 13 separate split-GAL4 lines that targeted different subsets of DNg02 cells with little or no expression in off-target neurons in addition to the one GAL4 line (GMR42B02) that targets 15 DNg02 cell pairs and the empty split-GAL4 line (SS03500) that serves as a control (Figure 3B). As a further test of the robustness of our results, this second round of optogenetic activation experiments was conducted under slightly different closed-loop visual conditions, in which the flies controlled the angular velocity of a single 18° dark stripe by regulating the difference in wing stroke amplitude between the two wings^14^. We altered the experimental conditions in this way because tethered flies typically fly more robustly when performing active stripe fixation. In this second set of optogenetic activation experiments, we observed an even stronger relationship between the change in wing stroke amplitude and the number of DNg02 cell pairs activated (slope = 2.77 degs per cell pair activated), as well as a higher correlation coefficient (r^2^ = 0.84) (Figure 3B).

To measure the changes in wingbeat amplitude plotted in Figure 3A and B, we subtracted the baseline level prior to activation from the peak response during activation. However, we also repeated the analysis using the measured peak response during activation without baseline subtraction (Figure S2). The slopes of the relationship determined with and without baseline subtraction were not significantly different for either the striped drum (p=0.59) or single stripe closed-loop (p=0.42) conditions.

We further explored the effects the DNg02 cells on wing kinematics by focusing on the time course of activation. Figure 3C plots the changes in wingbeat amplitude and stroke frequency for SS02625, a line that targets 8 DNg02 cells, which were collected during our second set of experiments using closed-loop conditions with a single stripe (Figure 3B). Whereas individual flies varied with respect to their background level of wingbeat amplitude prior to optogenetic activation (Figure 3B, top traces), the peak level of wingbeat amplitude typically reached the same approximate value for each fly during activation. This result suggests that the level of activation we applied elicited a saturating excitation of the DNg02 neurons within the lines. We also observed an intriguing pattern of changes in wingbeat frequency elicited by the excitation (Figure 3B, bottom traces). In most cases, DNg02 activation resulted in a correlated rise in both wingbeat amplitude and frequency, but in some instances the increase in amplitude was accompanied by a net decrease in frequency. Whether activation elicited a decrease or increase in wingbeat frequency was not random, but rather depended on the level of wingbeat frequency prior to activation. In particular, individuals that flew with a lower wingbeat frequency exhibited a large increase in frequency upon activation, whereas those that flew with a higher wingbeat frequency exhibited a decrease (Figure 3C). This trend was consistent across all the DNg02 lines, whether tested using a striped drum or a single stripe as the visual pattern for the closed-loop condition (Figure 3D, inset). A likely explanation for this peculiar trend emerges from plotting instantaneous wingbeat frequency against wingbeat amplitude throughout the time course of optogenetic activation for all the trials of an individual fly (Figure 3E). In each trial, CsChrimson activation evoked an initial rapid rise in both wingbeat amplitude and frequency; however, once the mean wingbeat amplitude of the two wings reached a value of about 160°, further increases in amplitude were accompanied by a decline in frequency (see also traces in the bottom panel of Figure 3C). Thus, when the DNg02 cells within the fly were maximally activated, we observed an inverse relationship between wingbeat frequency and amplitude.

The time-varying relationship between wingbeat amplitude and frequency (Figure 3D) bear a striking resemblance to data presented in a former study on the metabolic power requirements for flight^15^, in which changes in wingbeat amplitude and frequency were elicited by upward and downward visual motion rather than optogenetic activation of DNs. That study presented a model in which the mass specific mechanical power (P*_mech_) delivered by the flight muscles sustains the sum of induced power (the cost of lift) and profile power (the cost of drag) during flight. Profile power, which is the dominant term, is proportional to the product of the wingbeat frequency and amplitude cubed^3^ (see Star Methods for details). Thus, this model predicts that when the mechanical power generated by the flight muscles is constant—as it is when the asynchronous flight muscles are maximally activated—any increase in wingbeat amplitude must be accompanied by a decrease in frequency and vice versa. To analyze whether DNg02 activation may elicit near maximal power output, we superimposed isolines for mechanical power in the frequency-amplitude plane, using equations from the prior study^15^. The precise values of these isolines are only approximate, because they are based on average morphometric data for wing length, wing mass, body mass, and flight force of the flies used in the prior analysis^15^; we did not take those measurements on the flies used in our experiments. However, the salient observation is that the set of amplitude-frequency values elicited during peak DNg02 activation are bounded by the shape of a power isoline (P*_mech_~200 W kg^−1^), which thus enforces the observed inverse relationship between wingbeat frequency and wingbeat amplitude. These data suggest that the 100 ms optogenetic stimulus results in peak activation of the indirect flight muscle motor neurons that are driven by the 8 DNg02 neurons of this particular driver line (SS02625). We do not know, however, whether this activation elicited a maximum recruitment of the entire set of indirect flight muscles as it is possible that some power motor neurons are not targeted by the DNg02 cells within this line.

**Figure 4.**
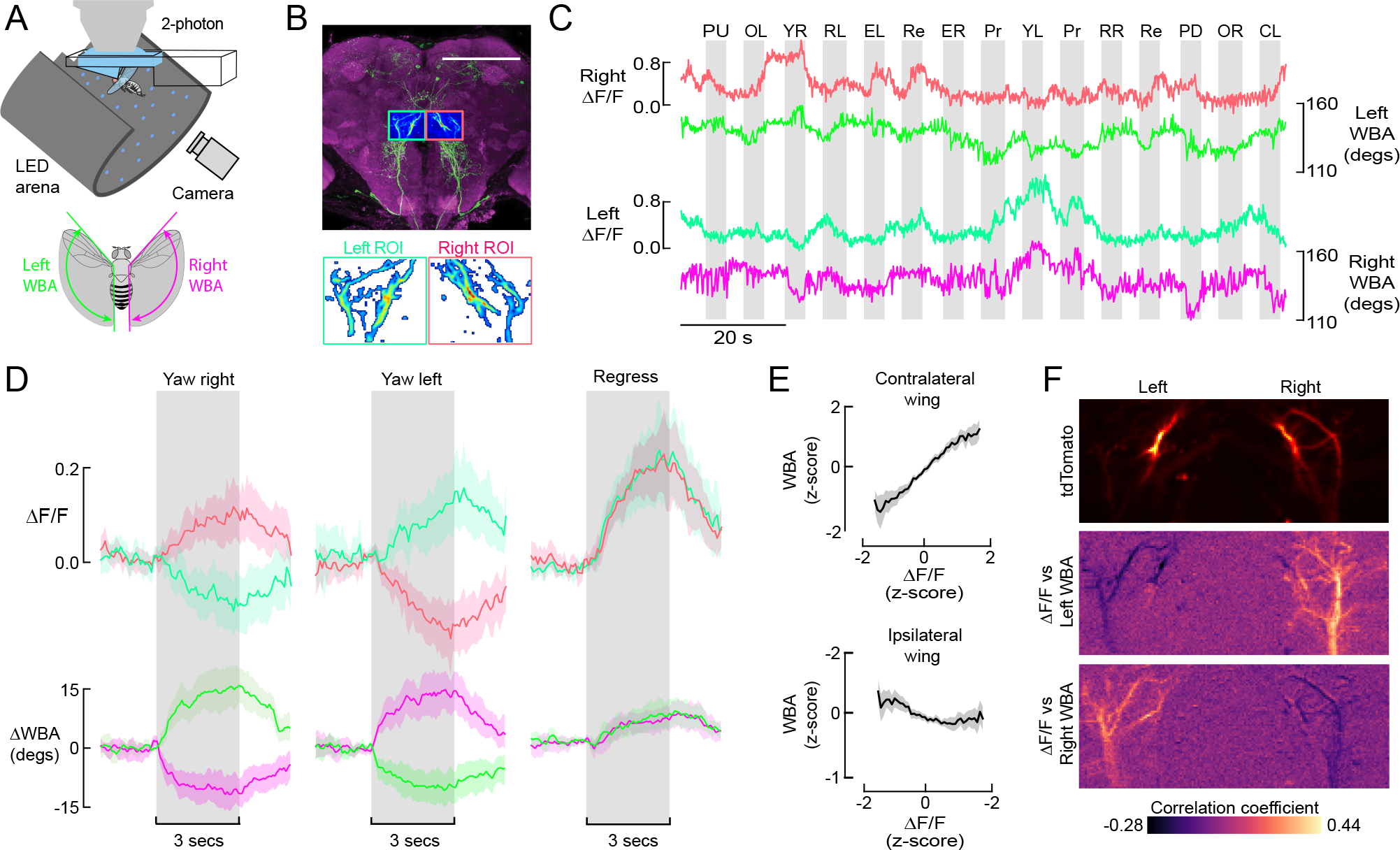
Left and right DNg02 neurons can act independently to regulate wingbeat amplitude. (A) Schematic showing fly tethered to a 2-photon microscope surrounded by an LED array for presentation of visual stimuli and a camera for tracking wing motion. Inset: a real-time machine vision system tracks wingbeat amplitude (WBA) the left (green) and right (red) wings of the fly. (B) During functional imaging, we captured DNg02 activity within left (green) and right (red) regions of interest, allowing us to measure simultaneous activity across populations of bilateral cells. The background image is replotted from Figure 2F. Lower inset: within the left and right ROIs, we used a standard deviation threshold to create a mask within which we measured changes in GCaMP6f fluorescence (ΔF/F) while the flying fly was subjected to different patterns of visual motion. (C) Example time traces of left and right DNg02 activity (measured as ΔF/F) along with changes in wingbeat amplitude during presentation of a panel of different visual stimuli (PU = pitch up, OL = stripe oscillating on left, YR = yaw right, RL = roll left, EL = expansion left, Re = regressive motion, ER = expansion right, Pr = progressive motion, YL = yaw left, RR = roll right, PD = pitch down, OR = stripe oscillating on right, CL = closed loop with stripe). (D) Averaged responses to three most informative patterns of optic flow: yaw right, yaw left, and regressive motion (N = 20 flies). See also Figure S3. Top row shows baseline-subtracted ΔF/F signals from left and right ROIs (green and red, respectively). Bottom row shows baseline-subtracted wingbeat amplitude signals for left and right wings (different shades of green and red, respectively). All data are presented as mean (solid line) and a boot-strapped 95% CI for the mean (shaded area); the 3-second period of stimulus presentation is indicated by the grey patch. (E) Wingbeat amplitude of the contralateral (top) and ipsilateral (bottom) wing plotted against ΔF/F. Data are derived from two-minute continuous flight recordings from 20 flies. Both fluorescence and wingbeat signals have been normalized to z-scores; data are presented as mean (solid line) and a boot-strapped 95% CI for the mean (shaded area). See also Figure S4. (F) Correlation between GCaMP6f fluorescence and wingbeat amplitude plotted on a pixel-by-pixel basis within the recorded ROI. The top image shows the expression pattern of tdTomato in the ROI of an example fly. The middle image shows the pixel-by-pixel correlation of the ΔF/F signal with left wingbeat amplitude recorded over a 2-minute flight bout; the bottom image shows the corresponding pixel-by-pixel correlation for right wingbeat amplitude. Note that activity in the DNg02 cells are positively correlated with wingbeat amplitude in the contralateral wing and negatively correlated with wingbeat amplitude of the ipsilateral wing.

So far, our activation experiments suggest that the population of DNg02 cells might function together to regulate flight power like a throttle, by controlling wingbeat amplitude in a bilateral fashion via symmetric activation of power and steering muscle motor neurons. However, this result may simply reflect the fact that optogenetic activation simultaneously excites both the left and right DNs in each fly. To test if left and right DNg02 cells might operate independently during steering maneuvers in flight, we performed 2-photon functional imaging from the dendritic region of the neurons in one of the DNg02 driver lines (SS02535) in tethered flying flies using GCaMP6f as an activity indicator (Figure 4A). We chose to focus on this particular line because: (1) we hoped that by being relatively sparse (3 cells) we would have the ability to resolve the signals in individual neurons (rather than record a spatially superimposed population response), and (2) this line happened to fly more robustly during functional imaging than many other lines we tested. In preparing the flies for recording, we dissected a window in the head capsule just dorsal to the esophageal foramen, which allowed us to image neurons on the left and right side of the brain simultaneously (Figure 4B). Because our goal was to test whether left and right DNg02 cells might be active independently, we subjected the flies to an array of different visual patterns during flight, chosen to elicit both symmetrical and asymmetrical wingbeat responses. These stimulus epochs included widefield patterns that simulated the optic flow experienced during pitch, roll, or yaw of the body, a lateral oscillating stripe, object expansion, progressive and regressive motion, and closed-loop stripe fixation (Figure 4C). The net results from this array of visual patterns was consistent in that they demonstrated that at least some DNg02 cells can respond differently on the left and right sides of the brain. This result was most apparent when the flies were presented with a yaw stimulus (Figure 4D, left traces), during which rightward motion elicited an increase in activity of the right DNg02 cells and a simultaneous decrease in activity of the left DNg02 cells (and vice versa for leftward motion). Responses to the full suite of open loop stimuli are presented in Figure S3. Although not all stimuli elicited strong responses in cell activity, the results in general support the inverse relationship between DNg02 activity and stroke amplitude of the contralateral wing. One noteworthy exception was the response to visual roll stimuli. In these cases, we recorded clear changes in the DNg02 GCaMP signals that were not mirrored by obvious alterations in stroke amplitude. Without further experiments, we have no clear explanation for this discrepancy with the responses to yaw, progressive, and regressive motion stimuli. However, the bilateral changes in wingbeat amplitude elicited by either mechanical or visual roll stimuli in tethered flight are much smaller than those elicited by yaw stimuli^16^. Further, an analysis on the aerodynamics of free flight maneuvers suggests that changes in the timing of angle of attack are much more critical for generating roll torque than changes in stroke amplitude^17^. One possibility is that during roll stimuli, the effects of DNg02 activity are antagonized by other descending commands that collectively act to maintain wingbeat amplitude at a near constant level. Testing this hypothesis will require the identification of more descending neurons types that are activated by wide field visual motion.

The changes in cell fluorescence were accompanied by the expected asymmetric changes in wingbeat amplitude for a yaw response. In contrast, bilaterally symmetrical visual patterns, such as regressive visual motion, elicited synchronous changes in the activity of the right and left DNg02 cells, accompanied by symmetrical changes in wingbeat amplitude (Figure 4D, rightmost traces). These results indicate that the DNg02 cells can operate independently on the left and right side of the brain in an asymmetrical or symmetrical fashion, depending on the pattern of visual input. Across all recordings, we measured a strong correlation between the DNg02 cells and the wingbeat amplitude of the contralateral wing, and a weaker anti-correlation with the wingbeat amplitude of the ipsilateral wing (Figure 4E). However, this weak negative correlation between the DNg02 cells and contralateral wing might simply result from the fact the wingbeat amplitudes of the two wings are negatively correlated (Figure S4). These patterns were readily apparent in individual recordings, in which we determined the correlation coefficient between changes in fluorescence (ΔF/F) and wingbeat amplitude for each pixel in the fluorescence image during a 20 second flight epoch (Figure 4F). A pixel pattern that corresponds to the arbor of the right DNg02 cells was highly correlated with left wingbeat amplitude, whereas a pixel pattern corresponding to the left DNg02 cells was highly correlated with right wingbeat amplitude.

## Discussion

In this paper, we describe a class of DNs in *Drosophila* (DNg02) that are unusual in that instead of existing as a unique bilateral pair, they constitute a large homomorphic population. By optogenetically driving different numbers of cells, we demonstrated that DNg02 cells can regulate wingbeat amplitude over a wide dynamic range (Figure 3A) and can elicit maximum power output from the flight motor (Figure 3D). Using 2-photon functional imaging, we also show that at least some DNg02 cells are responsive to large field visual motion during flight in a manner that would make them well suited for continuously regulating wing motion in response to both bilaterally symmetrical and bilaterally asymmetrical patterns of optic flow (Figure 4D).

Compared to birds, bats, and pterosaurs—the three other groups of organisms capable of sustained active flight—a unique feature of insects is that their wings are novel structures that are not modified from prior ambulatory appendages. Insects retained the six legs of their apterogote ancestors, but added two pairs of more dorsally positioned wings^18^. This evolutionary quirk has profound consequences for the underlying neuroanatomy of the insect flight system. Within their thoracic ganglia, the sensory-motor neuropil associated with the wings constitutes a thin, dorsal layer sitting atop the larger ventral regions that control leg motion^6,19,20^. Numerically, however, there appear to be comparable numbers of DNs targeting the wing and leg neuropils^6^. This is a bit surprising, given the more ancient status of the leg motor system and the importance of legs in so many essential behaviors. However, the relatively large number of flight DNs may reflect the fact that the control of flight requires greater motion precision because even minute changes in wing motion have large consequences on the resulting aerodynamics^21^.

Straight flight in *Drosophila* is only possible due to the maintenance of subtle and constant bilateral differences in wing motion, carefully regulated by feedback from sensory structures such as the eyes^22,23^, antennae^24,25^, and halteres^26,27^. The control system necessary for straight flight must permit the maintenance of very large, yet finely regulated, distortions of wing motion in order to produce perfectly balanced forces and moments. One means of controlling fine-scaled sensitivity over a large dynamic range is through the use of a population code with range fractionation, a phenomenon that bears similarity to the size principle of spinal motor neuron recruitment^28^.

The use of a population code to specify motor output is a general principle^13^ that has been observed in a wide array of species including leeches^29^, crickets^30^, cockroaches^31^, and monkeys^32^. In dragonflies, 8 pairs of DNs—a group of cells roughly comparable in number to the DNg02 cells—project to the flight neuropil and encode the direction to small visual targets^33^. In the case of the DNg02 cells, we hypothesize the that the population activity serves to trim out rotational torques and translational forces allowing the animal to fly straight.

Although the DNg02 neurons are morphologically similar, we strongly suspect that the population is not functionally homogeneous. To fly straight with perfect aerodynamic trim, an animal needs to zero its angular velocity about the yaw, pitch, and roll axes, in addition to regulating its forward flight speed, side slip, and elevation. Thus, if the DNg02 cells are the main means by which flies achieve flight trim, one would expect that they would be organized into several functional subpopulations, with each set of cells controlling a different degree of freedom of the flight motor system. For example, one subpopulation of DNg02 cells might be primarily responsible for regulating roll, while another is responsible for regulating pitch, and yet another regulates forward thrust. Such subpopulations need not constitute exclusive sets, but rather might overlap in function, collectively operating like a joystick to regulate flight pose. If this hypothesis is correct, we would expect the DNg02 neurons to differ with respect to both upstream inputs from directionally tuned visual interneurons as well as downstream outputs to power and steering muscle motor neurons. Unfortunately, we could not distinguish individual cell types across the different driver lines we used at the level of light-based microscopy. If DNg02 cells are further stratified into subclasses, it is likely that each driver line targets a different mixture of cell types. Indeed, the variation we observed in changes in wingbeat amplitude as a function of the number of DNg02 cells activated (Figure 3E) might reflect this variation in the exact complement of cells targeted by the different driver lines. Further, although one driver line (R42B02) targets 15 DNg02 neurons, it is likely that this number underestimates the size of the entire population, and we speculate that there may be a small set of neurons dedicated to regulating each output degree of freedom. Collectively, our results indicate that we have identified a critical component of the sensory motor pathway for flight control in *Drosophila,* the precise organization of which is now available for further study using a combination of genetic, physiological, and connectomic approaches.

## STAR METHODS

### RESOURCE AVAILABILITY

#### Lead contact

Further information and requests for resources and reagents should be directed to and will be fulfilled by the Lead Contact, Michael H. Dickinson (flyman@caltech.edu).

#### Materials availability

All new fly lines generated for this paper are listed in the key resources table.

#### Data and code availability

All data has been deposited on Mendeley at https://doi.org/10.17632/7g984jm2zc.1 and are publicly available as of the date of publication. The DOI is listed in the key resources table. All original code for data analysis has been deposited on Mendeley at https://doi.org/10.17632/7g984jm2zc.1 and is publicly available as of the date of publication. All original code used for the kinefly wing tracking software is publicly available on Github at https://github.com/ssafarik/Kinefly. The DOIs are listed in the key resources table. Any additional information required to reanalyze the data reported in this paper is available from the lead contact upon request.

### EXPERIMENTAL MODEL AND SUBJECT DETAILS

All experiments were conducted on genetically modified female *Drosophila melanogaster.* To create genetic lines that specifically targeted DNg02 cells, we used the split-GAL4 technique described previously (Luan et al. 2006; Namiki et al. 2018). We combined split half lines that have promoters to drive expression of either the transcription activation domain (p65ADZp) or the DNA-binding domain (ZpGAL4DBD) of the GAL4 protein. To identify driver lines containing DNg02, we manually searched the brain expression pattern from publically available GAL4 lines in the FlyLight database (https://flweb.janelia.org/). We chose pairs of these driver lines that appeared to target the DNg02 cells and screened the resulting split-GAL4 combinations by crossing them with flies carrying the reporter pJFRC200-10XUASIVS-myr::smGFP-HA inserted into attP18. For the optogenetic activation experiments, we used 3-to-6-day-old female flies obtained by crossing virgin females from each split-GAL4 line (or GMR42B02) with 3-to-5-day-old males carrying 20XUAS-CsChrimson-mVenus inserted into attP18. We reared the progeny of this cross on standard cornmeal fly food containing 0.2 mM all trans-Retinal (ATR) (Sigma-Aldrich) and transferred adult flies 0–2 days after eclosion onto standard cornmeal fly food with 0.4 mM ATR. We supplemented the standard cornmeal food with additional yeast. For functional imaging experiments, we used 2–5 days old female flies that resulted from a cross of the split-GAL4 line SS02535 with w+;UAS-tdTomato;UAS-GCaMP6f flies^35^.

### QUANTIFICATION AND STATISTICAL ANALYSIS

All experiments were analyzed with custom scripts written in either Matlab or Python. We set a value of p=0.05 as the threshold for a significant effect in this study. Sample sizes refer to the number of individuals tested. Variance across individuals is represented by either a standard deviation envelope, interquartile range, or boot-strapped 95% CI for the mean (Figure 1D, Figures 1E, 3A, 3B, and Figures 4D–E, S3B, S4A–C, respectively). For each of the two datasets used in Figure 3A and B, as well as Figures S2 and S3, we performed regression analyses with Minitab, under the assumptions of linearity, homoscedasticity, independence, and normality. For each of the two datasets in Figure 3A and B we compared the slopes of mean wing amplitude changes as a function of the number of DNs between baseline subtracted (BLS) and non-BLS wing amplitudes. For the two visual conditions in Figure 3A and B, the effect of the interaction term DN pairs * BLS mode did not affect the slope between the number of DN pairs and mean wing amplitude (striped drum and single stripe, p=0.59 and p=0.42, respectively).

### METHODS DETAILS

#### Optogenetic activation experiments

Female offspring of split-GAL4 driver lines crossed to flies carrying 20XUAS-CsChrimson-mVenus were anesthetized on a cold surface (4°C) and tethered to a tungsten pin with Loctite^®^ 3972^™^ UV-activated cement (Henkel). The tethered fly was positioned such that its stroke plane was horizontal and perpendicular to the vertical optical axis of a digital camera (Basler acA640-120 gm) equipped with an Infinistix 90-degree lens with a long-pass filter (LP715-30.5, Midwest Optical Systems). Two horizontally oriented fiber optic light guides (FT1500EMT; Thorlabs), each coupled to an IR light source (driver: LEDD1B, and LED: M850F2; Thorlabs) illuminated the stroke planes of the left and right wings. We used Kinefly software^9^ to track the anterior-most angular excursion of the fly’s left and right wingbeat (Figure 1C). Digital values for the left and right wingbeat amplitudes were converted into voltages using a PhidgetAnalog 1002 (Phidgets), and recorded on a Digitata 1440A data acquisition system (Axon Instruments) for subsequent analysis. The voltage signals from the two wings were also sent to an LED panel controller^14^ (IOrodeo) which was programmed so that flies could regulate the angular velocity of the visual display via the difference in wingbeat amplitude of the two wings. We used a custom-built photodetector circuit to record the oscillations in the incident IR light caused by the flapping motion of the wings and convert that signal into a voltage proportional to wingbeat frequency (https://github.com/janelia-kicad/light_sensor_boards), which we also recorded on the Digidata 1440A. For optogenetic activation, we positioned a fiber optic light guide (FT1500EMT; Thorlabs) beneath the fly, aimed at the thorax, which conducted the output of a 617 nm LED (M617F1, Thorlabs) at ~3.4 mW/mm^2^. The timing and duration of the pulse was controlled via the voltage input to the LED driver (LEDD1B, Thorlabs). The fly and ancillary instruments were surrounded by a 12 × 4 panel (96 × 32 pixel) LED arena (470 nm)^14^ that covered 216° of azimuth with a resolution of 2.25° in front of the fly. Our initial screen of 50 lines (Figure E1), was conducted under visual open-loop conditions. In each experiment, we elicited 30 responses to a 100 ms light pulse with an interpulse interval of 10 seconds. For each trial, we determined the average wingbeat amplitude (WBA) of the left and right wings over the 0.5 second period prior to stimulus onset and subtracted this value from the entire trace to create a zero baseline. The response to optogenetic activation was calculated as the average value of WBA over the 0.5 second period starting with stimulus onset. The 30 trials from each fly were averaged to create a single measurement for each individual. Our second screen, in which tested only lines that drive expression in DNg02, was identical to the first, except that we conducted the trials under visual closed-loop conditions, in which the difference in fly’s left-right wingbeat amplitude controlled the angular velocity of a striped drum with a spatial frequency of 36°.

#### Power Calculations

The mechanical power isolines presented in Figure 3E were based on a prior model that is described in detail elsewhere^15^, which in turn relied extensively on the calculations of Charles Ellington^3^. In this model, the animal’s thorax exhibits sufficient elastic storage to account for the inertial power required to accelerate the wings back and forth^36^, and muscle mass specific mechanical power, P*_mech_, is given by the sum of induced power, P*_ind_ (the cost of lift) and profile power, P*_pro_, (the cost of wing drag). Mass specific induced power is calculated as:

(1) $$P_{ind}^{*}={\left[\frac{F_{t}}{m_{M}g}\right]}^{\frac{1}{2}}{\left[\frac{F_{t}}{2\rho \text{Φ}R^{2}}\right]}^{\frac{1}{2}}\kappa$$

where *F*_t_ is total flight force, ρ is air density, Φ is wingbeat amplitude in radians, *R* is wing length, κ is the correction factor required because of the periodic nature of vortex shedding in the animal’s wake, *m*_M_ is the mass of the flight muscle and *g* is the gravitational constant. Mass specific profile power is given by:

(2) $$P_{pro}^{*}=\frac{\rho Sn^{3}{\text{Φ}}^{3}R^{3}{\hat{r}}_{3}^{3}(S){\bar{\left|d\hat{\phi }/d\hat{t}\right|}}^{3}}{16m_{M}g}\bar{C_{D,pro}}$$

where *n* is wingbeat frequency, *S* is the surface area of the two wings, ${\hat{r}}_{3}^{3}(S)$ is the dimensionless third moment of wing area, ${\bar{\left|d\hat{\phi }/d\hat{t}\right|}}^{3}$ is the mean cube of the absolute value of dimensionless angular velocity and $\bar{C_{D,pro}}$ is the mean profile drag coefficient. In some studies^3,15^, the mean profile drag, was estimated to be $\frac{7}{\sqrt{Re}}$, where Re is the Reynolds number of the flapping wing, which was calculated as:

(3) $$Re=\frac{s}{v}n\text{Φ},$$

where ν is the kinematic viscosity of air. The only terms in these equations that came from our own measurements were n and Φ, wingbeat frequency and wingbeat amplitude; all other parameters, including morphometric measures such as wing length and wing area were taken from the mean values for wild type *Drosophila melanogaster* measured previously^15^. Thus, the precise values for the isolines plotted in Figure 3E should be viewed with caution. Nevertheless, using these assumptions, the profile power term is much larger than the induced power term, which results in the hyperbolic shape of the isolines in Figure 3E, such that mechanical power is roughly proportional to (nΦ)^3^, predicting a strong inverse relationship between wingbeat amplitude and wingbeat frequency when the power delivered by the flight muscles is limited. Further, a previous study tested the $\frac{7}{\sqrt{Re}}$ approximation for mean profile drag, $\bar{C_{D,pro}}$, against direct measurements using a dynamically scaled flapping robot^37^ and found that the measured value based on the kinematics of a tethered fly (1.36) was substantially higher than the Re-based approximation (0.61), which suggests that profile power makes an even more dominant contribution to the total mechanical power requirements. This increases the expectation that instantaneous mechanical power will exhibit a dependence on wingbeat amplitude and frequency that is proportional to (nΦ)^3^. We used the higher, more accurate value for $\bar{C_{D,pro}}$ in our calculations (1.36), which is why our estimate for peak P*_mech_ (~200 W kg^−1^) is so much higher than that reported by Lehmann and Dickinson (77 W kg^−1^) ^15^.

#### Anatomy

To image expression patterns, we dissected the complete central nervous systems of 3-to-5-day-old female adult progeny in Schneider’s Insect Medium (Sigma), fixed them in paraformaldehyde, and then transferred them to a goat serum blocking buffer for 1 hr. We then replaced the buffer with the primary antibodies (mouse nc82 supernatant at 1:30, rabbit polyclonal anti-GFP at 1:1000) diluted in phosphate buffered saline with 0.5% Triton X-100 (PBT) and gently agitated the preparations for 36–48 hr at 4°C. After washing with PBT, the samples were then incubated with secondary antibodies (Alexa Fluor 488 goat anti-rabbit, and Alexa Fluor 568 goat anti-mouse at 1:400) diluted in PBT and agitated again at 4°C for 3 days. The samples were then washed, fixed again in paraformaldehyde, mounted on a poly-L-lysine cover slip, cleared with xylene, and embedded in dibutyl phthalate in xylene (DPX) on a microscope slide with spacers. After drying for two days, samples were imaged at either 20X or 40X with a confocal microscope (Zeiss LSM 510) (Dionne et al. 2018). To discriminate the morphology of individual DNg02 cells, we used a multi-color flip out technique^11^. To identify pre-synaptic terminal of DNg02, we examined neuronal polarity using a reporter (pJFRC51-3xUAS-Syt::smGFP-HA in su(Hw)attPa) that labels synaptotagmin, a synaptic vesicle-specific protein. More detailed descriptions of these protocols are available on the Janelia FlyLight website (https://www.janelia.org/project-team/flylight/protocols). All images are available through the FlyLight Split-GAL4 website (https://splitgal4.janelia.org/cgi-bin/splitgal4.cgi).

#### Functional Imaging

Tethered, flying flies were imaged at an excitation wavelength of 930 nm using a galvanometric scan mirror-based two-photon microscope (Thorlabs) equipped with a Nikon CFI apochromatic, near-infrared objective water-immersion lens (40x mag., 0.8 N.A., 3.5 mm W.D.). We used the 2–5 days old female progeny of a cross between the split-GAL4 driver line SS02535, which drives expression in 3 pairs of DNg02s and w+;UAS-tdTomato;UAS-GCaMP6f flies^35^. We recorded two channels to image tdTomato and GCaMP6f fluorescence in the posterior slope arbors of both left and right DNg02 neurons. Depending on the individual preparation, we acquired either 72 × 36 μm images with 128 × 64 pixel resolution at 13.1 Hz, or 72 × 29 μm images with 160 × 64 pixel resolution at 11.2 Hz. To correct for horizontal motion in the x-y plane, we registered both channels for each frame by finding the peak of the cross correlation between each tdTomato image and the trial-averaged image^38^. Based on tdTomato expression we selected a field of view (FOV) approximately centered along the medio-lateral axis. The 20% most variable pixels in the left and right halves of the GCaMP6f images were selected as the left and right regions of interest (ROIs) respectively. To correct for motion in z, we normalized the GCaMP6f fluorescence to the tdTomato fluorescence. For each frame and each side, we computed fluorescence (F_t_) of the GCaMP6f signal by subtracting the average of the background from the average of the ROI. The background was defined as the 20% dimmest pixels of the entire FOV. We computed the baseline fluorescence, F_0_, as the mean of the 10% lowest F_t_ in the ROI. To standardize the measured neuronal activity across individual preparations we normalized baseline-subtracted fluorescence to the maximum observed for each individual fly on each anatomical side of the brain as ΔF/F = (F_t_ − F_0_) / (F_t_ − F_0_) _max_. We presented visual stimuli with a 12 × 4 panel (96 × 32 pixel) arena that covered 216° of azimuth with a resolution of 2.25°, identical to that used in the optogenetic screen. To reduce light pollution from the LED arena into the photomultiplier tubes of the 2-photon microscope, we shifted the spectral peak of the visual stimuli from 470 nm to 450 nm by placing five transmission filters in front of the LEDs (one sheet of Roscolux no. 59 Indigo, two sheets of no. 39 Skelton Exotic Sangria, and two sheets of no. 4390 Cyan). We presented an array of visual patterns, each for 3 s, alternated with 3 s static starfields. The visual patterns were presented in a shuffled pseudo-random order and included roll, pitch, and yaw motion in both directions, a stripe oscillating on the left or right, an expanding object on the left or right, progressive and regressive motion, and closed loop stripe fixation. We illuminated the wings using four horizontal fiber-optic IR light sources (M850F2, Thorlabs) distributed in a ~90° arc behind the fly. We tracked left and right wingbeat amplitudes with a machine vision system, Kinefly^9^, at 32 Hz. This method introduces a delay in measurement of ~30 ms, which we corrected.

## Supplementary Material

2

## Figures and Tables

**KEY RESOURCES TABLE:** 

REAGENT or RESOURCE	SOURCE	IDENTIFIER
**Chemicals, Peptides, and Recombinant Proteins**		
All-trans-retinal	Sigma-Aldrich	CAS: 116-31-4
Schneider’s Insect Medium	Sigma-Aldrich	S0146
Triton X-100	Sigma-Aldrich	X100
Xylene	Thermo Fisher Scientific	x5-500
Dibutyl phthalate in xylene (DPX)	Electron Microscopy Sciences	13512
Paraformaldehyde	Electron Microscopy Sciences	15713-S
**Antibodies**		
Mouse mAb anti-Bruchpilot (nc82)	Developmental Studies Hybridoma Bank	nc82; RRID: AB_2314866
rabbit polyclonal anti-GFP	Thermo Fisher Scientific	Cat #: A-11122; RRID: AB_221569
Alexa Fluor 488 goat anti-rabbit	Thermo Fisher Scientific	Cat #: A-11034; RRID: AB_2576217
Alexa Fluor 568 goat anti-mouse	Thermo Fisher Scientific	Cat #: A-11031; RRID: AB_144696
**Deposited Data**		
Raw and analyzed data	This paper	https://doi.org/10.17632/7g984jm2zc.1
**Experimental Models: Organisms/Strains**		
D. melanogaster: UAS-CsChrimson	Bloomington Drosophila Stock Center	RRID:BDSC_55135
*D. melanogaster: UAS-OpGCaMP6f* (*20XUAS-IVS-Syn21-OpGCamp6F-p10* in *attP5*)	Gift from D. Anderson	N/A
*D. melanogaster: UAS-tdTomato* (*P{w[+mC]=UAS-tdTom.S}3*)	Bloomington	RRID:BDSC_36328
*D. melanogaster: UAS-OpGCaMP6f; UAS-tdTomato*	Constructed from above two lines	N/A
*D. melanogaster: pJFRC200-10XUASIVS- myr::smGFP-HA in attP18*	^34^	N/A
D. melanogaster: SS01074	Bloomington Drosophila Stock Center	RRID:BDSC_75840
D. melanogaster: SS01063	Bloomington Drosophila Stock Center	RRID:BDSC_75837
D. melanogaster: SS00735	Bloomington Drosophila Stock Center	RRID:BDSC_75998
D. melanogaster: SS01540	Bloomington Drosophila Stock Center	RRID:BDSC_75903
D. melanogaster: SS01052	Bloomington Drosophila Stock Center	RRID:BDSC_75825
D. melanogaster: SS01558	Bloomington Drosophila Stock Center	RRID:BDSC_75946
D. melanogaster: SS02377	Bloomington Drosophila Stock Center	RRID:BDSC_75874
D. melanogaster: SS02384	Bloomington Drosophila Stock Center	RRID:BDSC_75958
D. melanogaster: SS01053	Bloomington Drosophila Stock Center	RRID:BDSC_86728
D. melanogaster: SS02631	Bloomington Drosophila Stock Center	RRID:BDSC_75976
D. melanogaster: SS02536	Bloomington Drosophila Stock Center	RRID:BDSC_75940
D. melanogaster: SS01069	Bloomington Drosophila Stock Center	RRID:BDSC_75828
D. melanogaster: SS02542	Bloomington Drosophila Stock Center	RRID:BDSC_75941
D. melanogaster: SS01546	Bloomington Drosophila Stock Center	RRID:BDSC_75944
D. melanogaster: SS02392	Bloomington Drosophila Stock Center	RRID:BDSC_75878
D. melanogaster: SS1075	Bloomington Drosophila Stock Center	RRID:BDSC_75841
D. melanogaster: SS01056	Bloomington Drosophila Stock Center	RRID:BDSC_75818
D. melanogaster: SS01556	Bloomington Drosophila Stock Center	RRID:BDSC_75953
D. melanogaster: SS02393	Bloomington Drosophila Stock Center	RRID:BDSC_75933
D. melanogaster: SS02608	Bloomington Drosophila Stock Center	RRID:BDSC_75966
D. melanogaster: SS01058	^6^	N/A
D. melanogaster: SS02383	Bloomington Drosophila Stock Center	RRID:BDSC_75888
D. melanogaster: SS02552	Bloomington Drosophila Stock Center	RRID:BDSC_75942
D. melanogaster: SS02379	Bloomington Drosophila Stock Center	RRID:BDSC_75963
D. melanogaster: SS02635	Bloomington Drosophila Stock Center	RRID:BDSC_75969
D. melanogaster: SS03500	This paper	N/A
D. melanogaster: SS02111	Bloomington Drosophila Stock Center	RRID:BDSC_75935
D. melanogaster: SS02396	Bloomington Drosophila Stock Center	RRID:BDSC_75882
D. melanogaster: SS01579	Bloomington Drosophila Stock Center	RRID:BDSC_75902
D. melanogaster: SS02634	Bloomington Drosophila Stock Center	RRID:BDSC_75970
D. melanogaster: SS02617	Bloomington Drosophila Stock Center	RRID:BDSC_75967
D. melanogaster: SS01061	Bloomington Drosophila Stock Center	RRID:BDSC_75836
D. melanogaster: SS02551	This paper	N/A
D. melanogaster: SS02553	Bloomington Drosophila Stock Center	RRID:BDSC_75936
D. melanogaster: SS02627	This paper	N/A
D. melanogaster: SS01049	Bloomington Drosophila Stock Center	RRID:BDSC_75833
D. melanogaster: SS01541	Bloomington Drosophila Stock Center	RRID:BDSC_75891
D. melanogaster: SS01577	This paper	N/A
D. melanogaster: SS01578	This paper	N/A
D. melanogaster: SS01560	Bloomington Drosophila Stock Center	RRID:BDSC_75948
D. melanogaster: SS02535	This paper	N/A
D. melanogaster: SS01073	Bloomington Drosophila Stock Center	RRID:BDSC_75839
D. melanogaster: SS02625	Bloomington Drosophila Stock Center	RRID:BDSC_75974
D. melanogaster: SS01563	This paper	N/A
D. melanogaster: SS02630	This paper	N/A
D. melanogaster: SS02550	This paper	N/A
D. melanogaster: SS01562	This paper	N/A
D. melanogaster: GMR42B02	Bloomington Drosophila Stock Center	RRID:BDSC_68782
D. melanogaster: SS02624	Bloomington Drosophila Stock Center	RRID:BDSC_75972
D. melanogaster: SS02544	This paper	N/A
**Software and Algorithms**		
Kinefly wing tracking software	^9^	https://github.com/ssafarik/Kinefly
Python	https://www.python.org	RRID: SCR_008394
Matplotlib	https://matplotlib.org	RRID: SCR_008624
MATLAB	https://www.mathworks.com	RRID: SCR_001622
FIJI	https://fiji.sc/	RRID:SCR_002285
**Other**		
UV-activated cement	Henkel	Loctite® 3972TM
Digital camera	Basler	acA640-120 gm
Long-pass filter	Midwest Optical Systems	LP715-30.5
Fiber optic light guide	Thorlabs	FT1500EMT
IR light source LED	Thorlabs	M850F2
IR light source driver	Thorlabs	LEDD1B
Wingbeat analyzer	Phidgets	PhidgetAnalog 1002
Data acquisition system	Axon Instruments	Digitata 1440A
Panel controller	IO Rodeo	Panels display controller unit
Transmission filter	Indigo	Roscolux no. 59 filter
Transmission filter	Skelton Exotic Sangria	Rosco no. 39 filter
Transmission filter	Cyan	Rosco #4390 filter

## Highlights:

- An activation screen identifies a descending neuron that regulates wing motion
- This neuron, DNg02, exists as a large population of nearly homomorphic cells
- Collectively, the population regulates stroke amplitude over a large dynamic range
- Functional imaging indicates that DNg02 functions to control both steering and thrust
