# Supplementary material for: A population of descending neurons that regulate the flight motor of Drosophila
Source: Curr Biol. Author manuscript; Available in PMC 2022 Jun 19. (PMC9206711; doi:10.1016/j.cub.2022.01.008)
Supplement: 2 [file NIHMS1777912-supplement-2.pdf]

| Driver line | Cells targeted | VNC projection | | | Type | Cell pairs | DNg02 pairs |
|---|---|---|---|---|---|---|---|
| | | Dorsal | Ventral | Tectulum | | | |
| SS01074 | DNg07 | - | - | + | P | 4 | 0 |
| SS01063 | DNg28 | - | - | - | U | 1 | 0 |
| SS00735 | DNp16 | + | - | - | P | 3 | 0 |
| SS01540 | DNp09 | - | + | + | U | 1 | 0 |
| SS01052 | DNp25 | - | - | + | U | 1 | 0 |
| SS01558 | DNg26 | + | - | - | P | 1 | 0 |
| SS02377 | DNg15 | - | + | + | U | 1 | 0 |
| SS02384 | DNa04, DNa10 | + | - | + | U | 2* | 0 |
| SS01053 | DNp02 | - | + | + | U | 1 | 0 |
| SS02631 | DNb06 | - | + | - | U | 1 | 0 |
| SS02536 | DNa05 | + | - | + | U | 1 | 0 |
| SS01069 | DNg14 | - | + | + | U | 1 | 0 |
| SS02542 | DNb01 | + | + | + | U | 1 | 0 |
| SS01546 | DNa05, DNa07 | + | - | + | U | 2* | 0 |
| SS02392 | DNp18 | + | + | + | U | 1 | 0 |
| SS01075 | DNg29 | - | + | - | U | 1 | 0 |
| SS01056 | DNp28 | + | - | - | U | 1 | 0 |
| SS01556 | DNp15 | + | - | - | U | 1 | 0 |
| SS02393 | DNa08 | + | - | - | U | 1 | 0 |
| SS02608 | DNg12 | - | + | + | P | 4 | 0 |
| SS01058 | DNg09 | + | - | + | P | 5 | 0 |
| SS02383 | DNb01 | + | + | + | U | 1 | 0 |
| SS02552 | DNb03 | + | - | - | P | 2 | 0 |
| SS02379 | DNp02, DNp11 | - | + | + | U | 2* | 0 |
| SS02635 | DNg07, DNg08 | + | + | + | P | 10* | 0 |
| SS03500 | Blank control | - | - | - | U | 0 | 0 |
| SS02111 | DNg10 | + | - | - | P | 2 | 0 |
| SS02396 | DNb02 | + | - | - | P | 2 | 0 |
| SS01579 | DNg11 | - | - | + | P | 4 | 0 |
| SS02634 | DNg02 | + | - | + | P | 1 | 1 |
| SS02617 | DNg11 | - | - | + | P | 5 | 0 |
| SS01061 | DNg26 | + | - | - | P | 1 | 0 |
| SS02551 | DNg07 | - | - | + | P | 4 | 0 |
| SS02553 | DNp17 | + | - | - | P | 4 | 0 |
| SS02627 | DNg02 | + | - | + | P | 2 | 2 |
| SS01049 | DNp10 | - | + | + | U | 1 | 0 |
| SS01541 | DNa07 | + | - | + | U | 1 | 0 |
| SS01577 | DNg02 | + | - | + | P | 3 | 3 |
| SS01578 | DNg02 | + | - | + | P | 5 | 5 |
| SS01560 | DNa07 | + | - | + | U | 1 | 0 |
| SS02535 | DNg02 | + | - | + | P | 3 | 3 |
| SS02279 | DNg17 | + | + | - | U | 1 | 0 |
| SS01073 | DNg02 | + | - | + | P | 5 | 5 |
| SS02625 | DNg02 | + | - | + | P | 8 | 8 |
| SS01563 | DNg02 | + | - | + | P | 8 | 8 |
| SS02630 | DNg02 | + | - | + | P | 10 | 10 |
| SS02550 | DNg02 | + | - | + | P | 6 | 6 |
| SS01562 | DNg02 | + | - | + | P | 12 | 12 |
| R42B02 | DNg02, DNg03 | + | - | + | P | 36* | 15 |
| SS02624 | DNg02 | + | - | + | P | 8 | 8 |

**Table S1. Anatomical features of the 50 driver lines used in the activation screen, related to Figure 1.**
The lines in column 1 are listed according to the magnitude of the changes in wingbeat amplitude evoked by CsChrimson activation (as in Figure 1).
Column 2 lists the DNs targeted in each particular line. In addition to DNg02 and DNg03, the GAL4 driver line R42B02 also targeted several other DNs including DNg07, DNg08, and DNg09.
Columns 3, 4, and 5 indicate to which of the three broad VNC regions the neuron or neurons project (Dorsal, Ventral, or Tectulum). A '+' indicates projection to the indicated region; a '-' indicates the lack of projection.
Column 6 indicates whether the targeted cell is a unique- or population-type neuron ('U' or 'P'). DNg26 is labeled 'P', although the SS1558 line only labels one pair of this DN type. In the case of SS02635 (which targets two cell types) we marked the line as 'P' because DNg07 is a population-type neuron.
Column 7 indicates the number of cell pairs scored by counting somata as shown in Figure S1. For cases in which the driver line targets two or more DN types (marked by asterisks), the value indicates the total number of cell pairs.
Column 8 indicates the number of DNg02 cell pairs in each line.

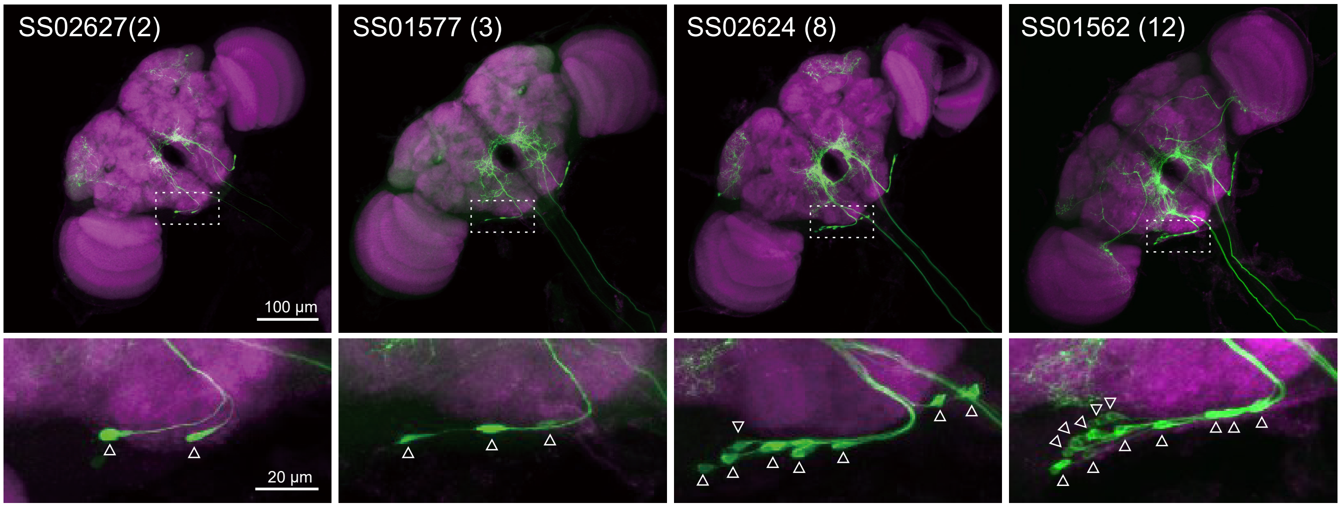


**Figure S1. The number of DNg02 neurons in each driver line may be determined by counting cell bodies, related to Figure 2.**

Maximum intensity projections for four of the DNg02 driver lines are shown with an inset indicating the region of the gnathal ganglion containing the cell bodies at higher magnification. The number of neurons targeted in each line is shown in parenthesis next to the line name. Membrane-targeted GFP expression is shown in green, nc82 staining is shown in magenta. White arrow heads point to individual cell bodies.

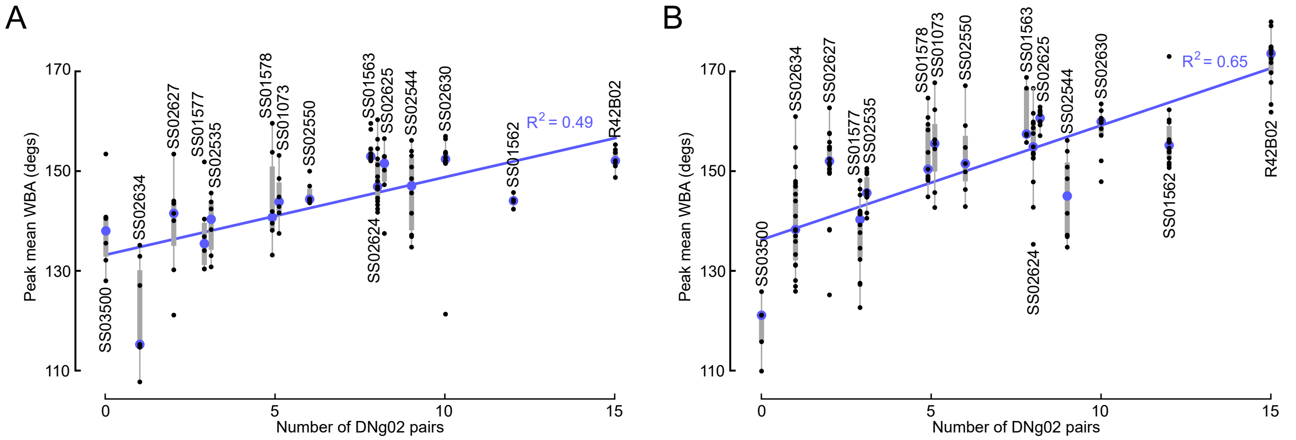


**Figure S2. The peak responses in mean wingbeat amplitude elicited by optogenetic activation are linearly correlated with the number of cell pairs in the DNg02 driver lines, related to Figure 3.**

These data differ from those plotted in Figure 3A and 3B in that the baseline wingbeat amplitudes prior to opogenetic activation were not subtracted from the measured changes elicited by activation. They thus represent the relationship between the absolute value of the peak wingbeat amplitude elicited by optogenetic activation and the number of cells in each line. The polling conventions are the same as those in Figure 3A and 3B.

(A) Data collected under visual closed-loop conditions using a striped drum; the slope of the regression is 1.6 degrees per cell pair activated ($r^2$= 0.49).

(B) Data collected under visual closed-loop conditions using a dark stripe; the slope of the regression is 2.3 degrees per cell pair activated ($r^2$= 0.65).

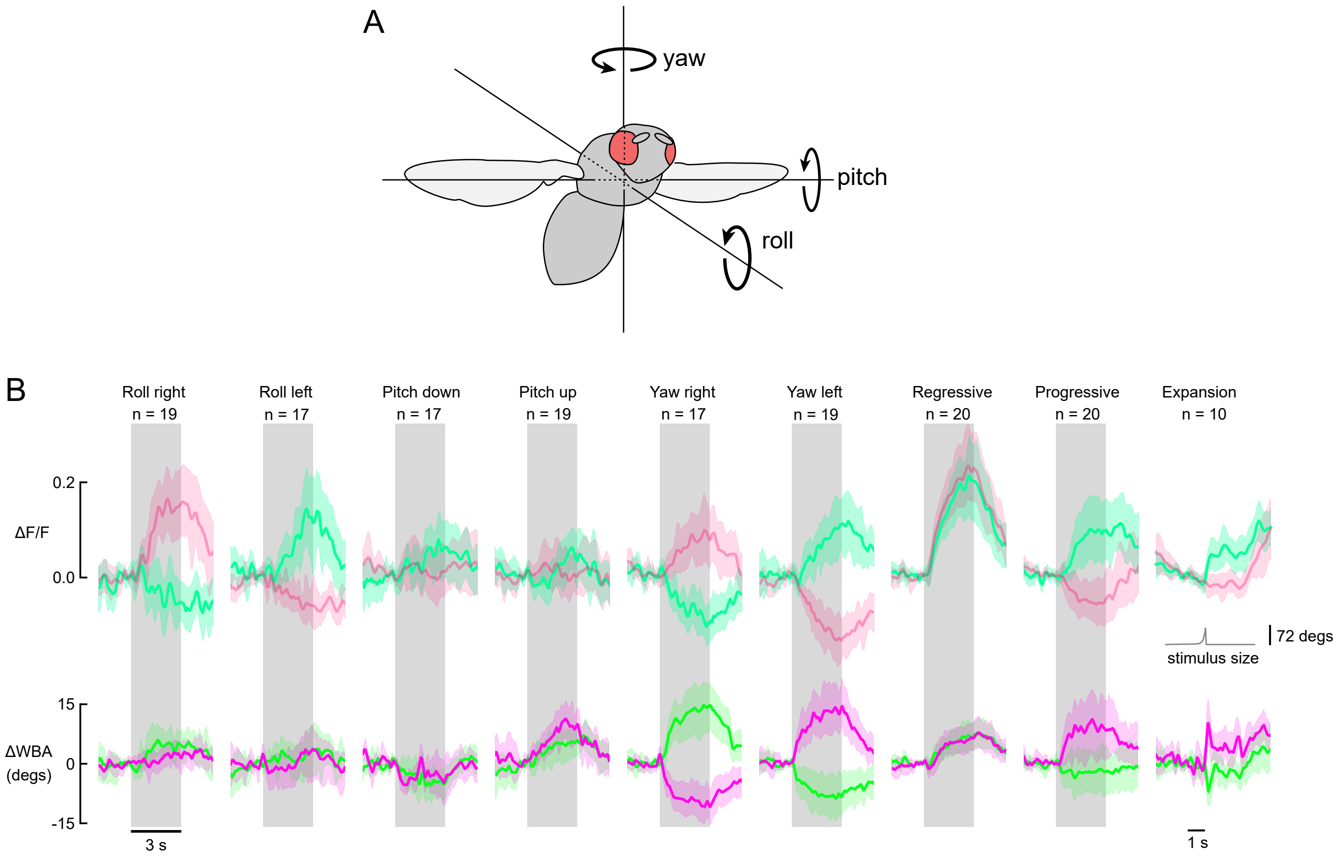


**Figure S3. Responses of DNg02 cells and wingbeat changes elicited by an array of different visual stimuli, related to Figure 4.**

(A) Cartoon illustrating the yaw, pitch, and roll axes for a fly in a normal flight posture. During functional imaging experiments, we presented moving starfield stimuli to create rotational visual motion around these axes, as well as translational stimuli that simulate forward motion (progressive) and backwards motion (regressive), and a dark expansion pattern that simulated a looming object.

(B) The physiological and behavioral responses during functional imaging experiments to 9 of the visual open-loop stimuli we presented to the flies. The gray areas indicate the duraton of the open-loop stimulis. We do not show the response to an oscillating stripe because we did not gather a sufficiently large sample size for that particular stimulus.

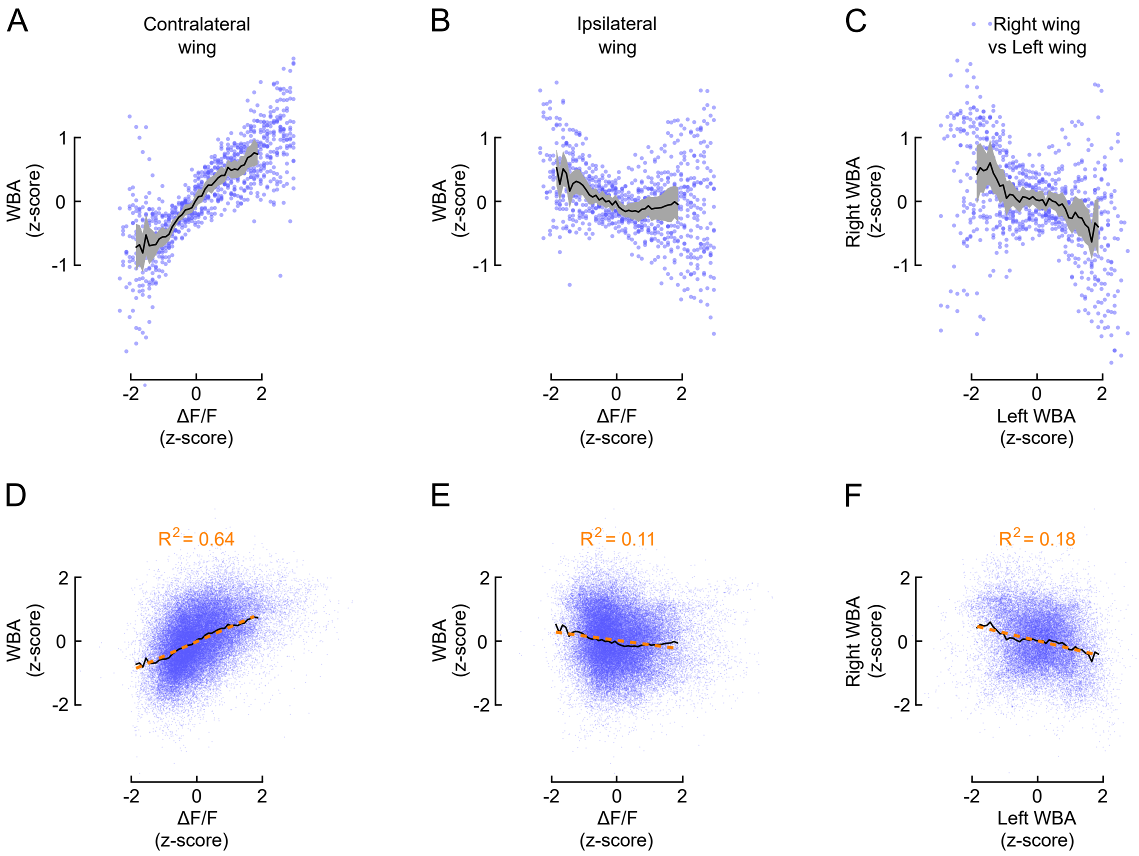


**Figure S4. DNg02 activity strongly correlates with contralateral wing angles, related to Figure 4.**
Data are derived from two, 2-minute flight recordings from each fly (n = 20 flies, driver line = SS02535). In panels A-C, we divided the abscissa into 36 bins, each with a width of 0.1 standard deviation (SD), and each data point shows the mean value of all points that fell within one of the 36 bins for each fly. The black lines indicate the individual mean wingbeat angle for 0.1 SD-wide bins of the normalized DNg02 fluorescence values of the 20 flies. The gray areas indicate the 95% confidence interval (CI) for the mean. The mean and CI data in panels A and B are replotted from Figure 4E.

(A) Individual cell fluorescence plotted against the amplitude of the contralateral wing, showing a strong positive correlation between contralateral wingbeat amplitude and DNg02 activity.

(B) Cell fluorescence plotted against wingbeat amplitude of the ipsilateral wing, indicating a weak negative correlation between the ipsilateral wingbeat amplitude and DNg02 activity.

(C) The left wingbeat amplitude plotted against the right wing beat amplitude indicates a clear negative correlation between the left and right wings.

(D – F) Similar to (A – C), but with all instantaneous values of wingbeat amplitude plotted as functions of the ΔF/F throughout the time series of the recordings. For comparison, we re-plotted the mean of the binned values from panels A, B, and C (black trace) along with a linear regression based on all the points (orange dashed line).

(D) For every SD increase of DNg02 GCaMP fluorescence, the contralateral wingbeat angle rises 0.45 SD ($p<0.01$, $r^2 = 0.64$).

(E) For every SD increase of DNg02 fluorescence the ipsilateral wingbeat angle falls 0.15 SD ($p<0.01$, $r^2 = 0.11$).

(F) For every SD increase of the left wingbeat angle, the right wingbeat angle decreases 0.15 SD ($p<0.01$, $r^2 = 0.18$). Such a negative correlation is expected, because the wingbeat amplitude of the two wings are anti-correlated whenever a fly executes a turn towards the left or right. This intrinsic correlation during all steering maneuvers could explain the weak negative trend in panels B and E.
